# Environmental sustainability, an essential outcome for implementation scientists to improve health

**DOI:** 10.3389/frhs.2025.1664446

**Published:** 2025-10-01

**Authors:** Virginia Mckay, Emmanuel Tetteh, Nicholas Wong, C. Bradley Kramer, Jason Burnham

**Affiliations:** ^1^ Center for Public Health Systems Science, School of Public Health, Washington University in St. Louis, St. Louis, MO, United States; ^2^ Washington University School of Medicine, Washington University in St. Louis, St. Louis, MO, United States; ^3^ Public Health–Seattle & King County, Seattle, WA, United States; ^4^ School of Public Health, University of Washington, Seattle, WA, United States

**Keywords:** environment, climate change, one health, implementation science, policy

## Abstract

While there is evidence of humans’ harmful impact on the environment, translating such evidence into changes is challenging. Implementation science can facilitate a shift from a reactive to proactive approach in tackling environmental sustainability. This article aims to spur further discussion among implementation scientists to incorporate environmental sustainability within their research, while also offering concepts relevant to environmental science researchers seeking to apply implementation science principles.

## Introduction

The evidence behind the impact of humans on the environment is overwhelming. One major impact is climate change, which refers to the long-term changes in temperature and weather patterns from human activity such as burning fossil fuels (e.g., coal and gas) which emit greenhouse gases (GHG) (1, 2). Global average temperatures have exhibited a clear upward trend, with the last 10 years being some of the warmest on record (1). This upward trend has been accompanied by seasonal weather extremes and an increasing frequency of natural disasters (3). Human activity also can harm the environment, for example, by releasing pesticides, pharmacological compounds that interrupt the normal biological functions and habitats of other living organisms (4, 5). Environmental harms and climate change have undeniable effects on human health––increases in frequency of extreme weather events, prevalence of vector-borne diseases, and disruptions to food production systems put human health and life at risk (6–8).

As we rapidly approach a threshold where the health of the environment and animal populations are at risk because of climate change and environmental degradation, it is difficult to anticipate whether our environment will continue to be inhabitable for our large human population (1). To adapt to, slow, or stop this change, environmental sustainability, in which we include both environmental and animal health, is of utmost importance. In response, there have been repeated calls among professional communities within all spheres to address environmental harms inflicted by human activity and promote a more sustainable environment (9, 10).

According to the United States' Environmental Protection Agency (EPA), striving towards sustainability means establishing and maintaining an environment where humans and nature can co-exist productively to support present and future generations (11). Many industries and scientific fields are incorporating environmental sustainability as a dimension for new interventions, products, and policies (12). Yet, the time lag between environmental health evidence and changes in practice and policy may be too long to sufficiently improve environmental health and slow climate change (13). Implementation science is the study of methods to promote the systematic uptake of research findings and evidence-based practices into routine practice, thereby improving the quality and effectiveness of health services (14–16). We argue implementation science is uniquely positioned to minimize this lag and provide structured processes that consider the context in which programs are applied and promote equitable uptake during implementation to maximize benefit (17). However, the field of implementation science largely has yet to engage in this issue.

In the following commentary, we give examples linking human health and environmental sustainability through the One Health framing. We note that implementation of the corresponding evidence-based approaches has been slow with mixed success. We then argue the role of implementation science to slow climate change and reduce its impacts on human health. Lastly, we make the case for a way forward by illustrating specific ways implementation science can support the effort to study, scale, and accelerate evidence-based interventions.

## The health of humans, animals, and the environment are intertwined

**Figure 1:**
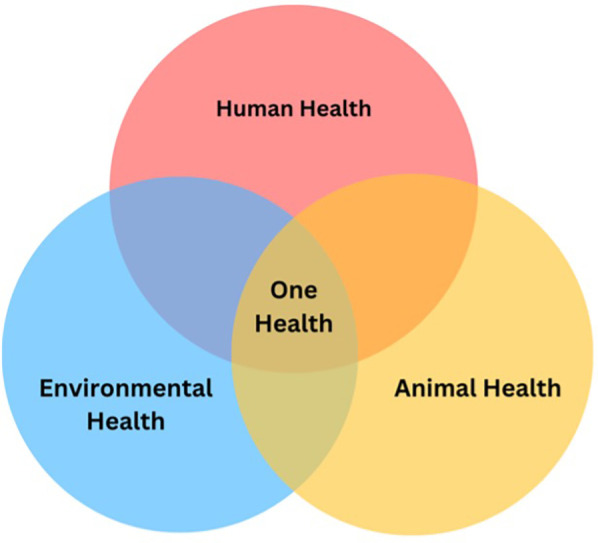
One health model adapted from CDC (18).

Incorporating environmental sustainability as a consideration in implementation science research and practice would represent a major paradigm shift. The One Health Model is a conceptual model gaining traction in scientific fields is being embraced by leading public health institutions globally such as the World Health Organization, the Centers for Disease Control and Prevention (CDC), and the Council on Education for Public Health accreditation criteria for schools of public health, prompting such a shift toward integrated approaches that address human, animal, and environmental health collectively (18–20). This simple model (Figure 1) suggests that the health of humans, animals, and the environment are inextricably linked, rather than separate entities and promotes “one health” where health is optimized for all three domains. It brings into relief how environmental and animal health can directly impact human health either in the short or long term. The model also suggests it is critical that we consider these other domains in balance with human health as the human population increasingly places stress on the other two domains.

## The impact of the environment on human health

Acknowledging the relationship between human health and environmental sustainability, there are at least two main mechanisms through which environmental sustainability, or a lack thereof, may affect population health relevant to implementation scientists: (1) by changing the intensity or frequency of health problems that people already face and (2) by creating new or unanticipated health problems in people or places where they have not been before (21). Conversely, efforts to maintain human health can have tremendous impact on environmental sustainability. We discuss each of these points in turn and provide examples.

### Changing the intensity or frequency of health problems

Six increasing major climate-driven event categories have been identified as key multi-pronged drivers of negative health impacts: floods, droughts, heatwaves, tropical storms, wildfires, and rising sea levels (22). For instance, growing evidence indicates that exposure to wildfire smoke leads to negative respiratory health effects, particularly exacerbating asthma symptoms (23–26). This is evident across various metrics such as hospitalizations, emergency department visits, and physician consultations (23, 27). Even modest environmental changes, such as small increases in temperature or shifts in precipitation, can heighten disease burdens. Vector-borne illnesses, defined as infections transmitted to humans through other animal carriers, often surge under conditions favorable to their carriers such as mosquitoes which carry malaria or tick borne diseases (23, 24, 24, 28–30).

Climate extremes also disrupt healthcare delivery by damaging infrastructure, interrupting medication supplies, and reducing access to care (27, 30). These disruptions compound other crises, for instance, during the COVID-19 pandemic, concurrent climate events increased susceptibility to infection, delayed emergency responses, and reduced system resilience. In the Amazon, endemic climate-sensitive diseases such as dengue further complicated COVID-19 detection and treatment (31, 32). The impact of these changes on human health has been significant, with projections indicating increasingly dire consequences. For instance, it is estimated that floods alone have the potential to cause 8.5 million deaths by 2050 (22).

### Creating new or unanticipated health problems in new places or populations

Unforeseen health complications are also arising from climate-related events. For example, a notable concern is the impact of wildfire smoke on birth outcomes in the United States. Low birth weight has been observed during wildfires in southern California, increasing risks of preterm birth (33, 34). Additionally, significant associations between smoke exposure and cases of gestational diabetes and gestational hypertension were recorded during multiple fire seasons in Colorado (22). The Chikungunya Virus carried by mosquitos, originally endemic to regions in Africa, emerged in Brazil in about 2010 and rapidly spread throughout Central and South America (35). Similar outbreaks and the virus' emergence in Europe, particularly in Italy and France, have been linked to the expansion of vectors carrying this virus due to meteorological extremes—rising temperatures and heavy rainfall in the affected regions (36, 37). These impacts extend to other types of infectious diseases including malaria, diarrheal illness, and fungal infections such as coccidioidomycosis (28, 30, 38, 39). Antimicrobial resistance and the rising ineffectiveness of existing antimicrobials have also been linked to climate change (40).

### Contributions of healthcare to environmental harm

Climate change and environmental impacts of human activity will continue to stress healthcare systems, yet healthcare systems and healthcare delivery simultaneously put stress on the environment and exacerbate climate change (27). In the US, the healthcare sector contributes approximately 9%–10% of all GHG emissions annually, with the major contributors being hospitals, outpatient clinical services, and pharmaceutical manufacture (41). Areas where excessive waste are ubiquitous and varied in many areas of healthcare, ranging from surgery, anesthesia, critical care, gynecology and infectious diseases as examples (42–45). The resulting impact on community health is an estimated loss of 400k disability-adjusted life years (DALYs) due to healthcare pollution (46). Much of the impacts (82%) is attributed to indirect emissions, defined as the supply chain of goods and services used for healthcare (46). Healthcare systems also produce significant amounts of waste including plastics, food waste, metal, and glass (47, 48). Estimates suggest that plastics constitute approximately 60% of hospital waste, including hard plastics such as syringes, and soft plastics such as protective masks (47). While some of this waste will decompose in landfills, some waste produced by the healthcare system must be sorted and disposed of separately, like radioactive or a biologically hazardous material (49). Radioactive materials must then be stored in locations, often underground, to prevent exposure. Similarly, biologically hazardous or “biohazardous” waste, which can contain infectious agents, must also be disposed of properly, often through incineration. Yet studies suggest that clinicians often do not properly sort materials and that both biohazardous materials are mixed in with general waste and general waste is mixed in with biohazardous material (47). When biohazardous waste is mixed in with general waste, it serves as a potential exposure pathway for infectious agents. Conversely, if general waste is mixed with biohazardous waste, GHG emissions are unnecessarily increased because biohazardous materials are typically incinerated.

## Slow and mixed success with efforts to address environmental sustainability

Growing acknowledgement that current impacts of human behavior on environmental and animal health are unsustainable has led to major environmental sustainability movements globally, as well as the development of interventions to promote environmental sustainability. However, the implementation of those interventions has been inconsistent and often lacking evidence. For example, the U.S. Department of Health and Human Services Health Sector Climate Pledge commits participating healthcare organizations to reducing greenhouse gas emissions and increasing climate resilience (50, 51). While the pledge signals a strong commitment, reporting and action has been uneven (52, 53). Similarly, Practice Greenhealth offers tools, benchmarking, and support for sustainable healthcare operations, but adoption remains inconsistent across facilities, highlighting the gap between available resources and widespread implementation (53, 54).

Similar to efforts to mitigate the impact of global warming and environmental harms at large, there is a general movement among professional organizations within healthcare to begin considering and reducing the impact of the system on environmental sustainability with multiple potential evidence-based approaches to reducing the carbon footprint and environmental harms of healthcare (55–57). Among some of the studied areas of healthcare to improve environment sustainability include the use of anaesthetic gases and other materials in various settings including cataract, hand, and orthopedic surgeries (42, 43, 58, 59). Perhaps one of the most well documented health interventions appropriate for targeting is metered dose inhalers (MDIs). MDIs are used to treat several respiratory illnesses including asthma and chronic pulmonary disease, but also contain hydrofluorocarbons which contribute more than 1,000 times the warming potential as the equivalent amount of carbon dioxide (60). Dry powder inhalers offer an alternative by delivering the same needed medications while replacing hydrofluorocarbons, yet their prescription is variable. In the UK, it is estimated that MDIs make up 70% of prescriptions while in Sweden MDIs only make 14% of prescriptions (61). Once used, MDIs must also be disposed of properly, primarily through incineration, to prevent gas leakage into the atmosphere. However, estimates suggest that approximately 70% of individuals dispose of inhalers in the general waste (62). Beyond this single example, multiple collections of evidence-based interventions are available or in development at various points within the healthcare system to support environmental health while maintaining human health. These include improving the efficiency of pharmaceutical manufacturing, reducing excessive waste in healthcare systems (including reducing the use of unnecessary or low-value care), decreasing the use of plastics and single use items, and lowering energy consumption (63, 64).

## A way forward with implementation science

**Figure 2:**
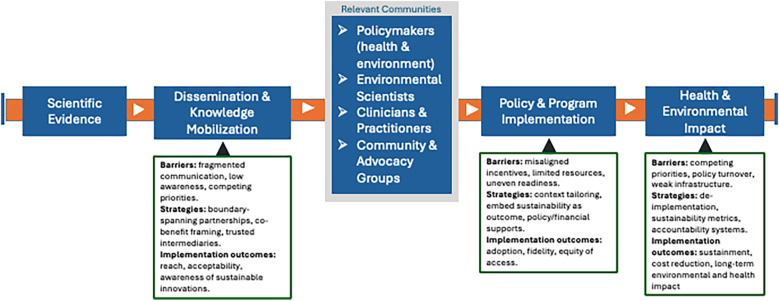
Environmental sustainability dissemination and implementation pipeline model. [Adapted from Farrelly et al. (62)].

We argue that implementation science has a role to play in fostering environmental sustainability and its relationship with human health, and implementation scientists should consider environmental sustainability in their research. Evidence-based interventions exist at scales ranging from international policy to individual-level action, and there have been some efforts within implementation science to address environmental sustainability, such as promoting telehealth or de-implementing harmful, unnecessary, or low-value care (64). Yet there are a paucity of studies applying dissemination and implementation science to implement climate mitigation and adaptation strategies, or trials of health interventions that proactively integrate environmental sustainability. Figure 2 adapted from Ferrelly and colleagues (65) illustrates key stages in the process of translating scientific evidence into practical benefit from the identification of an innovation, actively disseminating the innovation, engaging with relevant communities, going through the implementation process, and realizing the ultimate impact. We have adapted it to incorporate aspects of environmental sustainability and common ways that implementation science might influence this process.

We assert that it is precisely a proactive approach, a central principle of dissemination and implementation, that is needed to address this global issue rather than expecting that passive integration of environmentally sustainable measures will be sufficient. Simultaneously, there is growing interest within fields that are newer to implementation science, like environmental health for example, that traditionally examines the impact of environmental exposures on human health, to leverage implementation science to promote environmental sustainability and human health (13). With this in mind, implementation scientists can support this active process by applying expertise and skill sets to these problems. Points of interaction between environmental sustainability and implementation science might be: (1) using innovations or evidence-based practices developed in environmental science to inform the selection of and leveraging implementation science principles to guide implementation, (2) including environmental sustainability as an implementation outcome, and (3) integrating scientific knowledge of environmental sustainability into adaptation, sustainment, and de-implementation processes for healthcare.

Our earlier examples highlight the many ways that implementation scientists can engage in research supporting environmental sustainability as well as the multiple ways that challenges in environmental sustainability intersect with relevant questions in implementation science. Although not comprehensive, we pose several questions in the endeavor to promote implementation science engagement in environmental sustainability that simultaneously align with current frontiers in implementation science:.

### What are the determinants and strategies that promote environmental sustainability in conjunction with human health?

Several studies suggest that clinicians as well as patients and communities are interested in more sustainable healthcare practices, but often do not know what better approaches are available or how to implement them (66, 67). Beyond individual determinants such as awareness of an environmentally more sustainable alternative, it is unclear the additional determinants that impact failed implementation for any given intervention. One example intervention with upstream barriers to implementation is a governmental initiative by U.S. State-led Boards of Pharmacy, which partner with drug disposal companies to provide opportunities for safe disposal of unused or unwanted medications. These programs specify eligibility criteria that limits which organizations are eligible for the program such as a licensed pharmacy or a narcotic treatment center (68). This may pose a barrier to individuals who wish to properly dispose of their medications but may not have easy access to these organizations. Implementation scientists hold a wealth of expertise on how to identify critical implementation determinants and can support this identification process.

Within the field, effective dissemination strategies continue to be underdeveloped, and we suggest that environmental sustainability interventions with implications for human health have the potential to serve as a test ground for developing effective dissemination strategies. Presumably implementation strategies are also needed to successfully integrate environmentally sustainable innovations in routine practice. Developing and testing strategies that would foster adoption and implementation of environmental sustainability interventions could help support the evidence-based for strategies at large.

### How can and should interventions be adapted and sustained to promote environmental sustainability while maintaining human health in a global context?

Researchers within the implementation science field recognize that maintaining fidelity to interventions is useful, but that adaptation to local context and circumstances is essential to ensure that interventions are successfully implemented and maintained. Climate change mitigation measures, for example, will presumably require tailoring to the diverse communities globally that must deploy them. Collaboration with environmental scientists and local communities will be essential ensuring continued intervention effectiveness in local contexts. By way of example, indigenous knowledge systems provide models of such contextual responsiveness. Concepts such as Two-Eyed Seeing, which integrates Indigenous and Western knowledge, and Three-Eyed Seeing, which explicitly incorporates the land, offer valuable perspectives for integrating evidence-based intervention to foster environmental sustainability (69, 70).

Implementation scientists are uniquely positioned to help support this process given that collaboration and engagement are staples of the field. However, there are new concepts emerging in these fields relevant to adaptation and fidelity, like concept of adaptive capacity which acknowledges that the mere existence of adaptation options does not guarantee implementation success in various communities or settings (71). Burton and colleagues outline six critical determinants of adaptive capacity: economic resources, technology, information and skills, infrastructure, institutional support, and equity (71). They emphasize that identifying and enhancing these determinants within a community or system is essential for reducing vulnerability to environmental harm and ensuring the effective implementation of adaptation options and strategies. While these insights are promising, current research often overlooks the fidelity of adaptation strategies and lacks robust methods to measure these determinants (72). This gap presents a significant opportunity for implementation scientists to advance the field by developing comprehensive frameworks and assessment tools tailored to climate change adaptation strategies. There is also a need to emphasize that adaptation is not a new activity solely relevant in the context of climate change, but an ongoing process aimed at reducing vulnerability to both natural climate variability and human-induced climate change.

Closely related to adaptation within implementation science is the concept of intervention sustainment and whether interventions can be sustainably implemented. Within the field, we typically consider intervention sustainment over time to be beneficial for human health. We have yet to reconcile the concept of sustainability in implementation science with environmental health.

### What strategies effectively reduce or eliminate healthcare that is unnecessary or wasteful?

The field of de-implementation within implementation science examines interventions that are ineffective or harmful and evidence-based approaches for removing these interventions from wide-spread use in practice. It is estimated that as much as 30% of all healthcare intervention may be unnecessary, and there are also well-documented examples of ineffective intervention in public health (73, 74). The harm caused by excessive intervention to human health and wasted healthcare resources is well documented, but the environmental impact of unnecessary intervention is not well understood. If examined, the documentation of environmental impacts could lend additional motivation for eliminating unnecessary intervention. Furthermore, implementation researchers are currently endeavoring to develop strategies that support both the effective removal of unnecessary intervention and replacement of more effective or efficient interventions (75). As with the example of MDIs, effective strategies that target policy, clinicians, patients, and caregivers to foster the replacement of MDIs with dry powder inhalers alone would make significant progress toward reducing GHG emissions produced through the health system. Given the natural alignment of environmental sustainability and implementation science researchers in the field of de-implementation, there is tremendous potential for implementation scientists to consider and influence the environmental impact of healthcare.

### Are there other outcomes that we should be considering as implementation scientists to understand environmental impacts?

In this work, implementation scientists may find it helpful to draw on the expertise of environmental scientists when selecting appropriate measures and interpreting results. Central to incorporating environmental impacts in implementation science studies is the approaches to conceptualizing and measuring these impacts. The One Health model suggests some ways that this may be possible, especially as an outcome, namely the environmental impacts of intervention implementation as they relate and are intertwined with human health outcomes. In some cases, the environmental impacts may be contributing to the health outcome of interest, such as in the case of fires exacerbating asthma. In other cases, the interrelatedness of factors may make the problem more dynamic as in the case of COVID and extreme weather events. For investigators that may be interested in this field, there are approaches to help document environmental impacts.

One such tool is the Greenhouse Gas Equivalencies Calculator (GGEC) developed by the EPA. The GGEC is a system that allows users to input either energy or emissions data to quantify the amount of carbon dioxide produced. Additionally, the GGEC provides more familiar, equivalent reference data such as the number of gallons of gasoline consumed that equates to the same amount of carbon dioxide emitted. The GGEC also provides a sustainability equivalence component which details how energy or emissions usage can be offset, for example, by carbon sequestration from a certain number of acres of forests (76). Another related tool is the M+ Waste Care Calculator, which is targeted toward healthcare waste managers, allowing them to calculate the environmental impacts of certain waste disposal routes depending on factors such as the type of waste, the disposal method, and the waste quantity (77).

A third tool is the Life Cycle Assessment (LCA), which can shed light on the environmental impacts of products from its stages of raw material to final disposal. LCAs are useful in quantifying the environmental impacts of commonly used items. For example, in a hospital intensive care unit (ICU), the electricity for mechanical ventilators and GHG emissions and pollutants produced from plastics for syringes can be quantified. LCAs are particularly useful in helping to examine the entire life cycle of a product in search of opportunities to reduce its environmental impact (78). All these tools can be used to provide scientists with quantitative data to record environmental impacts.

### What challenges may implementation scientists face?

Implementation scientists are well positioned to advance the ideas presented here, drawing on the expertise of environmental scientists. It is also important to acknowledge the limits of what it can achieve. Some within the implementation science field might suggest that focusing on environmental sustainability draws attention away from the goal of implementation science, which has historically been improving human health. Yet, we argue this is a false distinction given that benefits to environmental health are ultimately to the benefit of human health either in the short or long term. The concept of balancing optimal health does not necessarily mean ignoring opportunities to improve human health. We can and should continue to engage in efforts to improve human health.

Certain barriers may persist regardless of improvements in implementation strategies. For example, misaligned financial incentives can pose a significant obstacle. While some sustainability interventions can generate cost savings or be cost-neutral, others require substantial upfront investment or ongoing costs, making them less likely to be pursued even when evidence of their health and environmental benefits is strong. Health system priorities present another challenge. The primary mission of most healthcare organizations is to deliver safe, effective, and timely patient care. Environmental sustainability, though increasingly recognized as important, often competes with urgent operational or clinical demands and may be deprioritized when perceived to conflict with these core responsibilities.

These constraints emphasize the value of targeting implementation science toward contexts where it can have the greatest effect initially, specifically, interventions that already have support, fit within organizational priorities, and are feasible given existing resources. In such situations, evidence-based implementation strategies can help close the gap between intent and practice, enabling the routine adoption of sustainable practices without compromising patient care, and build support for an implementation science approach. Implementation scientists will likely need partners in other sectors to motivate change similar to areas of health and healthcare where implementation scientists have been successful. Lastly, this commentary is written primarily with implementation scientists in mind; however, many of the considerations here are equally relevant to policymakers, health system leaders, environmental scientists, and community stakeholders, all of whom play essential roles in advancing sustainable practices that safeguard both human and planetary health.

## Conclusion

As the threat of climate change continues to escalate, there is a compelling opportunity for dissemination and implementation scientists to actively engage and explore ways to address this urgent issue. Effective interventions to address environmental sustainability only effectively mitigate exposures and prevent diseases if they are efficiently disseminated, adopted, implemented, and sustained. Dissemination and implementation science can play an important role in translating climate plans into actionable strategies and outcomes. Furthermore, there may be opportunities for implementation research to enhance resilience to climate change by integrating environmental sustainability into the design and implementation of health interventions.

## Data Availability

The original contributions presented in the study are included in the article/Supplementary Material, further inquiries can be directed to the corresponding author.
